# Experimental Study on Mechanical Performance of Basalt Fiber-Reinforced Polymer Plates with Different Bolted Connection Configurations

**DOI:** 10.3390/polym17192627

**Published:** 2025-09-28

**Authors:** Zhigang Gao, Dongzi Pan, Qing Qin, Chenghua Zhang, Jiachen He, Qi Lin

**Affiliations:** 1 School of Civil and Architecture Engineering, Xi’an University of Science and Technology, Xi’an 710000, China; gaozhigang@xust.edu.cn (Z.G.); qinqingjd@163.com (Q.Q.); zch-0819@163.com (C.Z.); 2 School-Enterprise Joint Research, Center of Underground: Structure Earthquake Resistance, Shaanxi-Province “Four Main Bodies and One Joint”, Xi’an 710054, China; 3 Xi’an XD New Energy Co., Ltd., Xi’an 710000, China; hjc08802022@163.com (J.H.); linqi@xd.cee-group.cn (Q.L.)

**Keywords:** BFRP plates, bolted connections, mechanical performance, sensitivity analysis, bearing capacity prediction model

## Abstract

Basalt fiber-reinforced polymer (BFRP) composites are increasingly utilized in photovoltaic mounting systems due to their excellent mechanical properties and durability. Bolted connections, valued for their simplicity, ease of installation, and effective load transfer, are widely employed for joining composite components. An orthogonal experimental design was adopted to investigate the effects of key parameters—including bolt end distance, number of bolts, bolt material, bolt diameter, preload, and connection length—on the load-bearing performance of three bolted BFRP plate configurations: lap joint (DJ), single lap joint (DP), and double lap joint (SP). Test results showed that the DJ connection exhibited the highest average tensile load capacity, exceeding those of the SP and DP connections by 45.3% and 50.2%, respectively. This superiority is attributed to the DJ specimen’s longer effective shear length and greater number of load-bearing bolts. Conversely, the SP connection demonstrated the largest average peak displacement, with increases of 29.7% and 52.9% compared to the DP and DJ connections. The double-sided constraint in the SP configuration promotes more uniform preload distribution and enhances shear deformation capacity. Orthogonal sensitivity analysis further revealed that the number of bolts and preload magnitude significantly influenced the ultimate tensile load capacity across all connection types. Finally, a calculation model for the tensile load capacity of bolted BFRP connections was established, incorporating a friction decay coefficient (α) and shear strength (τ). This model yields calculated errors under 15% and is applicable to shear slip-dominated failure modes, thereby providing a parametric basis for optimizing the tensile design of bolted BFRP joints.

## 1. Introduction

Traditional photovoltaic (PV) mounting systems primarily utilize metal materials such as aluminum alloy and stainless steel. However, their significant drawbacks—including high self-weight, insufficient corrosion resistance, and relatively high coefficients of thermal expansion (CTEs)—severely limit their long-term performance in harsh environments like hot-humid and salt-fog conditions [1,2]. Basalt fiber-reinforced polymer (BFRP) composites, characterized by lightweight properties, UV resistance, chemical inertness, and low thermal conductivity, have emerged as an ideal material for next-generation PV mounting systems. They can effectively reduce structural self-weight by 30–50% while significantly extending service life to over 30 years [3,4,5].

Given that the effective transmission of loads heavily relies on reliable connections, the complexity of engineering structures and connection nodes inevitably impacts their widespread adoption. Common connection configurations for composite materials include adhesive bonding, riveting, and bolted connections [6,7]. Bolted connections, serving as the primary load-transfer mechanism and structural interface for advanced composite structures, are critical for overall structural safety and stability [8]. Bolted connections offer unique advantages through standardized interface design: Preload control enables optimized stress distribution at the connection interface [9,10], and modular assembly characteristics significantly enhance construction efficiency [11].

Current research offers abundant findings on connection parameter analysis for composite structures. Lin Qingyuan et al. [12] analyzed assembly parameter uncertainty in composite bolted connections, established a bearing capacity prediction model and reliability assessment methodology, and optimized assembly parameters to enhance structural reliability. Zhao Shuyuan et al. [13] studied the tensile properties and failure behavior of ceramic matrix composite (CMC)/super alloy double-bolt connections under high-temperature conditions, revealing the influence mechanism of geometric parameters on connection performance. Shi Jianwei et al. [14] established a finite element model (FEM) to reveal the influence of structural parameters on load distribution unevenness and connection strength in CFRP hybrid adhesive-bolted joints. Zuo Y. [15] combined a synergistic damage mechanics approach with FEM to elucidate the correlation mechanism between matrix crack propagation and stiffness degradation in CFRP/Ti bolted joints, finding that preload torque can enhance joint stiffness and suppress crack propagation. Li, X. et al. [16] conducted a detailed parametric study on key parameters influencing the failure strength of multi-bolt composite joints using a fracture mechanics-based numerical method. Olmedo Á. [17] established a 3D progressive damage model for countersunk bolt single-fastener joints in composite laminate panels. FEM results were compared with experimental data to analyze tensile properties, showing good agreement. Quang T.D. et al. [18] experimentally validated the reinforcing effect of glass fiber sheet (GFS) reinforcement (woven fabric or chopped strand mat) on PGFRP multi-bolt connections, finding that bearing capacity could be increased by up to 177%. Chybiński [19] conducted experimental studies on the load-slip behavior of aluminum-wood composite bolted connections reinforced with toothed plates, analyzing the effects of bolt diameter (10 mm and 12 mm) and bolt grade (5.8 and 8.8) on connection behavior. Sun et al. [20] investigated the relationship between bolt preload and bolt hole bearing resistance using FEM and experimental methods, finding that preload has little effect on connection strength but significantly influences failure mode.

In practical analysis, isolating a single factor for independent analysis is often infeasible; instead, the coupled effects between various factors and target parameters must be comprehensively considered [21]. To ensure the safety and reliability of composite bolted connections, parameter sensitivity analysis must be performed on composite connection structures, uncertainties must be quantified, and design robustness must be enhanced [22]. In this study, an orthogonal experimental design was employed to reveal the influence patterns of parameters—including bolt material, end distance, number of bolts, and preload—on the failure modes and bearing capacity of BFRP plate joints under different connection configurations. The significant factors affecting ultimate bearing capacity and peak displacement were identified, and parameter allocation was optimized. Based on experimental results, a bearing capacity calculation model incorporating the friction decay coefficient (α) and shear strength (τ) was established to provide a reference for engineering design.

### 1.1. Experimental Setup

**Figure 1:**
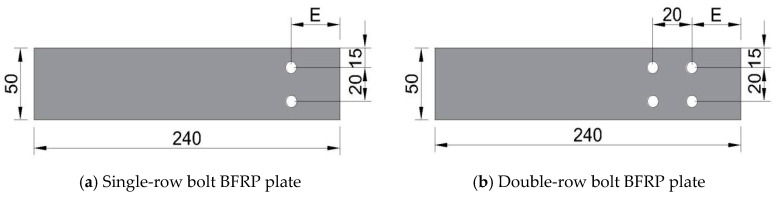
Test specimen size diagram (unit: mm).

**Figure 2:**
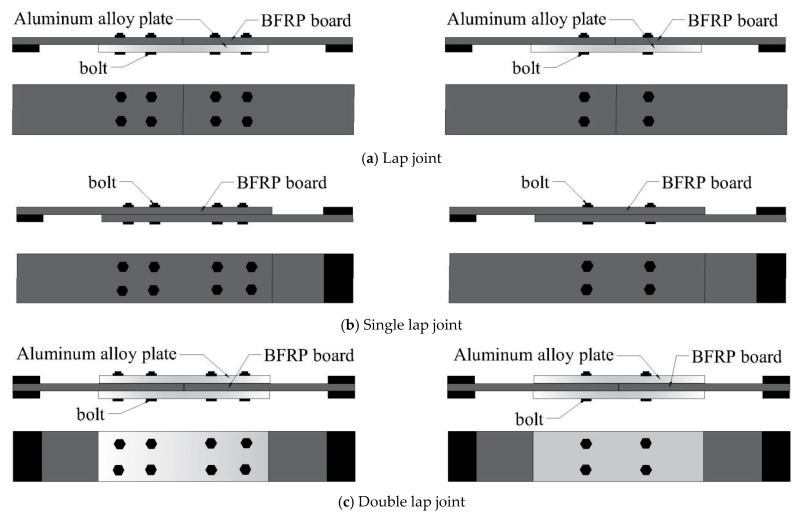
Various bolt joint configurations for BFRP plates.

This experiment employed an L8 orthogonal design, with the factor levels shown in Table 1. The BFRP plates used for connection were manufactured by Pavoco Composites Co., Ltd. (Huaying, China), with dimensions as shown in Figure 1. The test focused on three different bolt connection forms (as shown in Figure 2), namely lap joint (DJ), single lap joint (DP), and double lap joint (SP).

Eight sets of specimens were prepared for each connection configuration based on the orthogonal table (Table 2). For each connection configuration, three replicate specimens were fabricated and tested to evaluate the variability of the experimental results, totaling twenty-four sets of specimens. The specimens consist of BFRP plates, bolts, aluminum alloy plates, and end reinforcement plates, with the length of the aluminum alloy plate serving as the connection length.

### 1.2. Physical and Mechanical Properties of Materials

The mechanical properties in Table 3 and Table 4 were provided by Pavoco Composites Co., Ltd., (Huaying, China) and were measured in strict accordance with ASTM D3039 [23] and other relevant standards under standard laboratory conditions. The BFRP plate employs a balanced and symmetric [0°/90°] layup sequence. Table 5 shows the aluminum alloy sheets used in tensile tests.

### 1.3. Specimen Preparation

**Figure 3:**
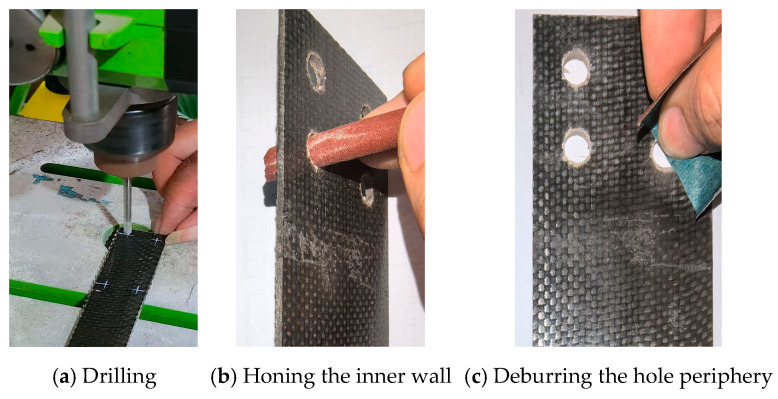
Specimen preparation.

A high-precision bench drill press was utilized for the drilling process. The BFRP panel was securely mounted, together with a wooden backup board, onto the worktable using clamps. A carbide-tipped core drill was employed as the drilling tool. The operation strictly adhered to the principle of “high rotational speed (1500 RPM) and low feed rate” (Figure 3a).

Upon completion of drilling, the dust was thoroughly cleaned from the hole using compressed air. Subsequently, the burrs on the hole wall and edges were removed by gently rotating a pencil wrapped with fine-grit sandpaper within the hole. This process resulted in a clean, defect-free hole (Figure 3b,c).

### 1.4. Loading Test Plan

#### 1.4.1. Loading Device and System

**Figure 4:**
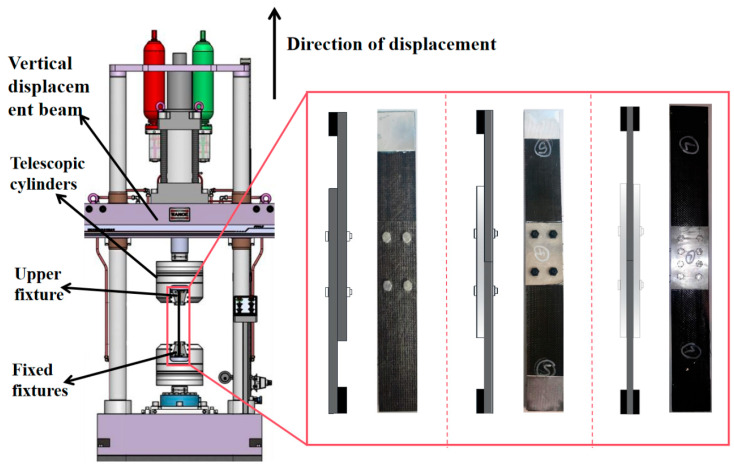
Tensile test loading.

The loading device used in the test was an HDT series 255 A electro-hydraulic servo fatigue testing machine for uniform loading (Figure 4), with a maximum test force of 300 kN.

#### 1.4.2. Test Procedure

Tensile testing was performed in accordance with the ASTM D5961 Standard Test Method for Bearing Response of Polymer Matrix Composite Laminates [24], with certain modifications as detailed herein. The principal aim of this study was to evaluate the ultimate failure load and failure modes of the bolted connections, rather than to obtain precise material-level bearing stress-strain behavior.

To minimize parasitic bending moments, specimens were securely clamped using serrated, self-aligning grips. The longitudinal axis of each specimen was carefully aligned with the loading direction along the centerline of the grip assembly prior to tightening. Relative displacement (slip) between the connected plates was determined based on crosshead displacement measurements.

Although ASTM D5961 recommends the use of an extensometer for quantifying localized bolt-hole deformation, crosshead displacement was considered a sufficient and reliable measure for the objectives of this investigation for the following reasons.

(1) The study emphasizes comparative performance assessment—specifically, ultimate load and peak displacement—across different connection configurations under identical testing conditions. Crosshead displacement provides a consistent basis for such comparisons.
(2) The failure modes of interest (e.g., shear slip, bearing failure) involve large, global displacements that are adequately captured by crosshead motion.
(3) System compliance was accounted for via prior calibration, and the high stiffness of the testing frame and fixtures relative to the specimens ensured that recorded displacements primarily reflected deformation within the specimen connection.
(4) A constant crosshead displacement rate of 2 mm/min was applied, which falls within the range (1–5 mm/min) permitted by the standard.
(5) An initial preload of 1.5 kN was applied to ensure proper seating of the specimen within the grips and to eliminate any initial slack or misalignment.

## 2. Test Results and Analysis

### 2.1. Lap Joint (DJ) Specimen Failure Mode

**Figure 5:**
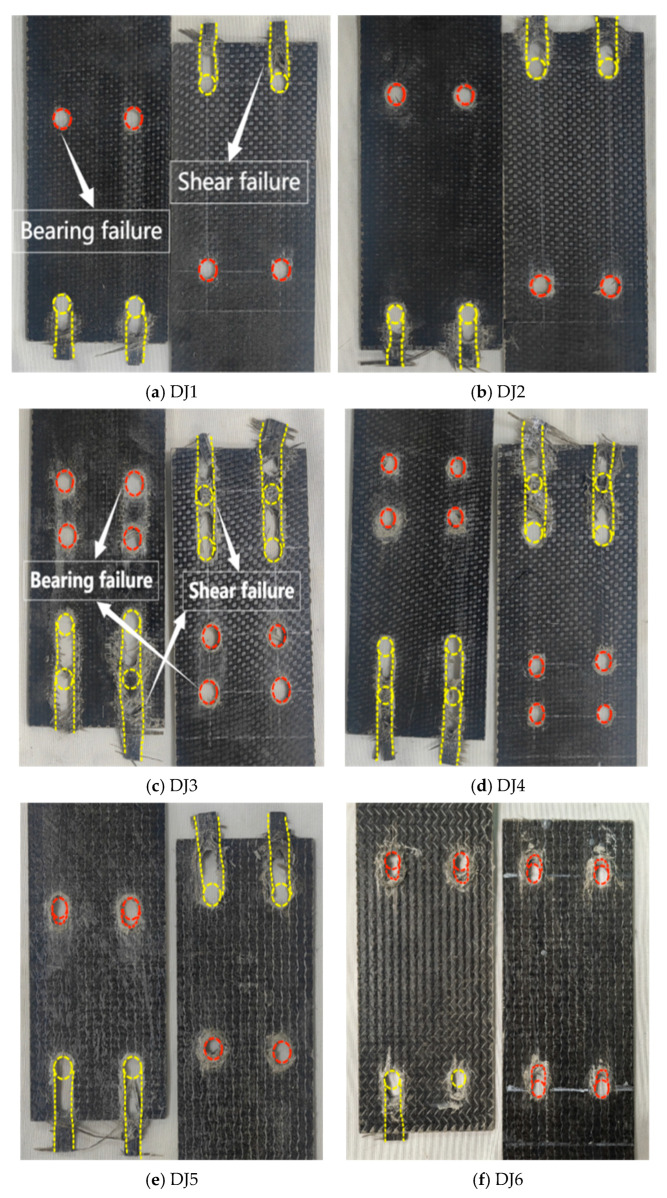

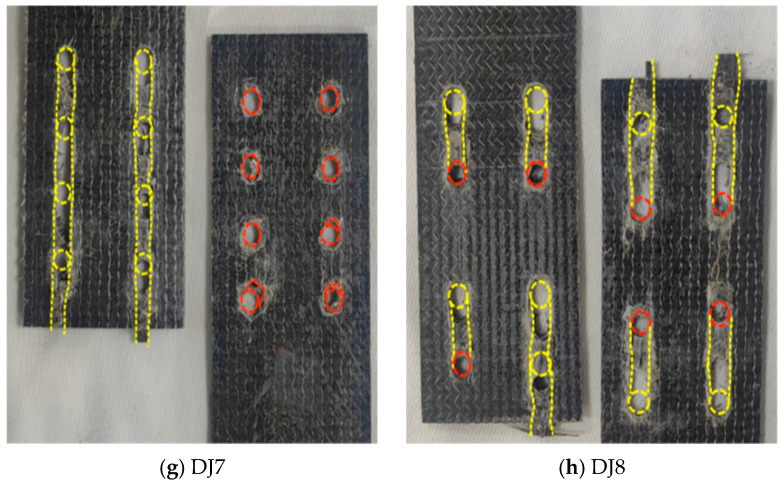
Failure mode diagram of the lap joint test specimen BFRP sheet (The red dashed line represents bearing failure, while the yellow dashed line represents shear failure).

The lap joint (DJ) specimens consistently exhibited shear failure (yellow dashed line in Figure 5) and compression failure (red dashed line), with nearly identical failure progression: Failure initiated in the drilled hole region and propagated along the fiber direction. Primary load transfer occurred through the bolts, which contacted the hole walls and induced compressive stresses. This generated tensile stresses on both sides of the hole [25], while shear stresses dominated the compression-tension transition zone. Stress concentration at the hole edges increased structural vulnerability. Additionally, drilling severed fibers at the hole location, reducing local load-bearing capacity. As loading increased (15% of the ultimate load), the structure reached its shear limit first, initiating shear failure and crack formation. Further loading propagated cracks along the path of least resistance—the unreinforced longitudinal direction—ultimately resulting in combined shear-compression failure. Specimens DJ3, DJ4, DJ7, and DJ8 displayed the most severe shear failure, with DJ7 exhibiting continuous longitudinal shear between holes. Larger bolt diameters (DJ2, DJ3, DJ5, DJ8) reduced hole-edge stress concentration through increased bearing area, producing more pronounced compression damage and shear slip zones. In contrast, smaller bolt diameters (DJ4, DJ7) intensified localized compression at hole edges due to concentrated pressure, exacerbating continuous shear failure of bolt holes and matrix cracking [26]. In low-preload specimens (DJ3, DJ6), reduced friction load transfer shifted more load to bolt-hole-wall contact. This intensified compressive forces, worsening matrix cracking and fiber delamination.

### 2.2. Single Lap Joint Specimen (DP) Failure Mode

**Figure 6:**
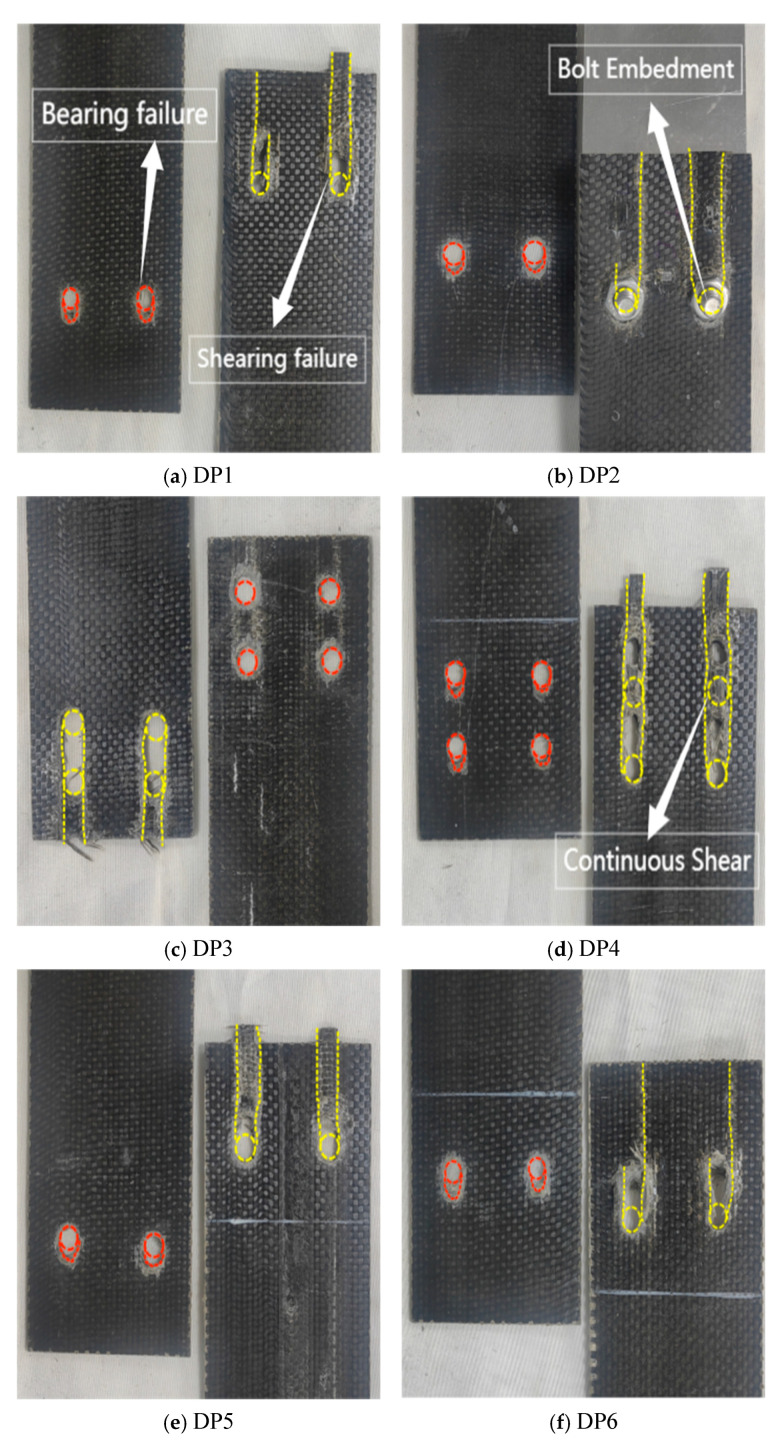

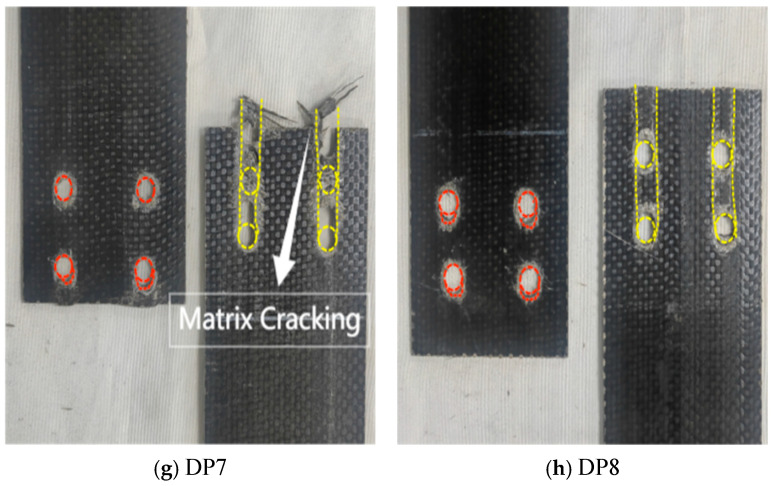
Single lap joint test specimen BFRP sheet failure mode (The red dashed line represents bearing failure, while the yellow dashed line represents shear failure.

**Figure 7:**
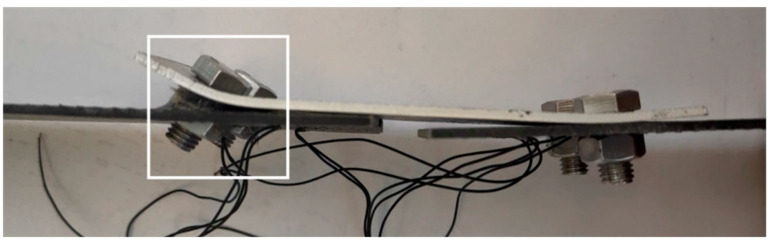
Nut flipped and inserted into screw hole (DP2).

The single lap joint specimens exhibited bolt rotation and embedment into the bolt holes, with the failure mode being a combination of compression and shear failure (Figure 6). The failure process occurred as follows: During the initial loading phase (approximately 15% of the ultimate load), the edge of the hole at one end of the BFRP plate was compressed by the bolt, producing audible compression fractures. This damage was potentially caused by asymmetric tensile loads. As the load increased (to approximately 65% of the ultimate load), the plate material near the bolt hole underwent significant bending and entered compression yielding. Simultaneously, the material near the bolt hole reached its shear strength first, resulting in shear failure. As shear failure progressed under continued loading, the BFRP plate experienced significant bending due to asymmetric loading. The 6 mm-diameter bolt exhibited noticeable tilting, while the nut flipped and embedded into the bolt hole (Figure 7). Compared to specimens with 6 mm bolt diameters, specimens with 10 mm bolt diameters featured a larger bearing area at the hole-wall interface, resulting in more uniform stress distribution. Consequently, the plate material near the bolt hole remained relatively intact, preventing nut embedment and reducing bolt tilting. Specimens DP2 and DP6 exhibited longer connection lengths, which contained shear failure and prevented propagation to the material ends. Increased bolt diameter reduced local stress at hole edges through larger bearing areas. Specimens DJ2, DJ3, DJ5, and DJ8 consequently showed more pronounced compression damage and shear slip zones. Conversely, specimens DJ4 and DJ7, with smaller bolt diameters, experienced concentrated pressure on the hole walls. This intensified compression stresses near hole edges, causing severe continuous shear failure of bolt holes and matrix cracking.

### 2.3. Double Lap Joint Specimen (SP) Failure Mode

**Figure 8:**
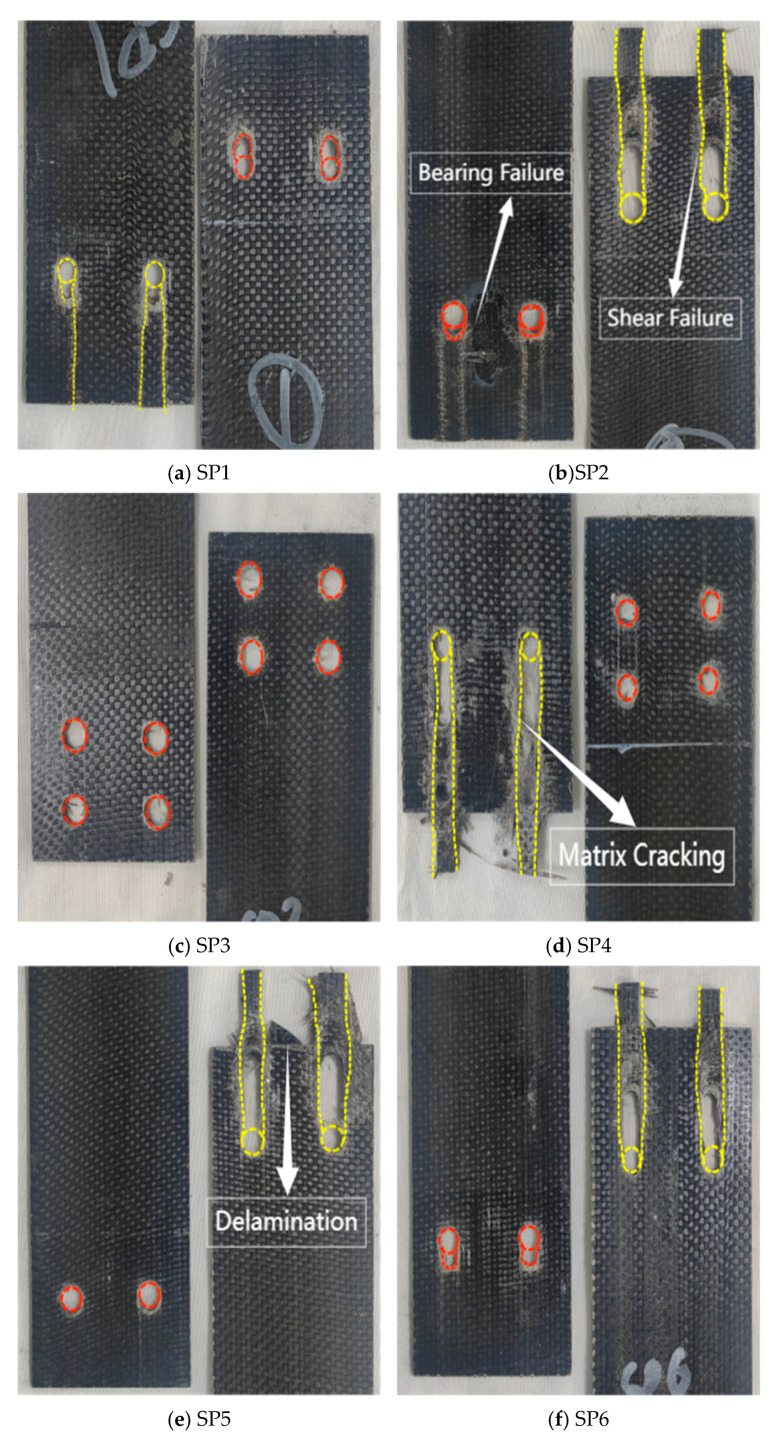

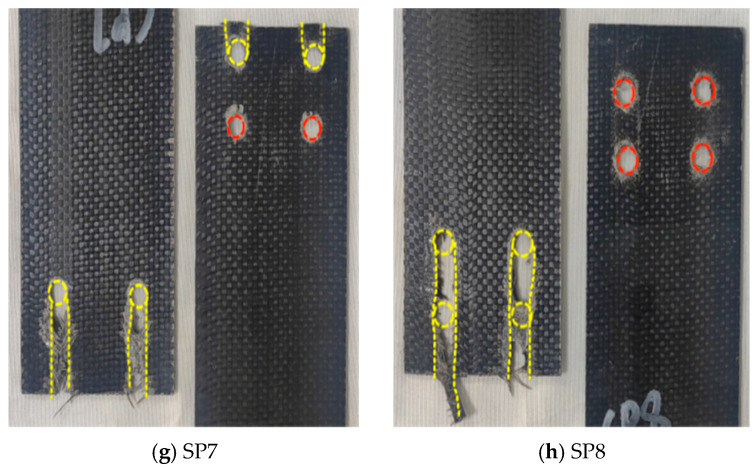
Failure mode of the double lap joint test specimen BFRP sheet (The red dashed line represents bearing failure, while the yellow dashed line represents shear failure).

The load transfer in double lap joint specimens was symmetrical, which eliminated load eccentricity and the resulting additional bending stresses. Due to the constraints imposed by the aluminum alloy plates, the material could only undergo shear failure along the fiber distribution direction, with accompanying severe matrix cracking and fiber delamination (Figure 8). All eight specimens exhibited shear slippage along the fiber direction extending to the material end. All double-row bolt group specimens exhibited continuous shear failure between holes (Figure 8d,g). The most severe damage occurred at the bolt holes, where the bolts and holes maintained maximum contact throughout the loading process. This caused the bolts to squeeze the hole walls and continue slipping.

#### SEM Electron Microscope Scanning Results

**Figure 9:**
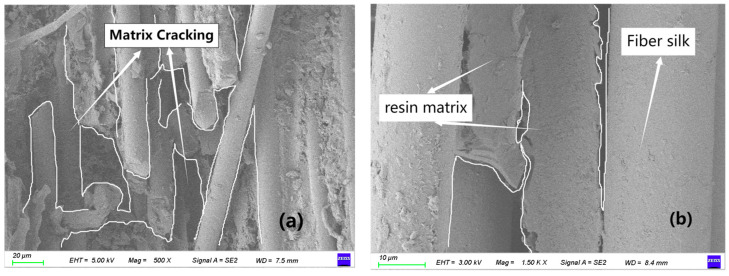

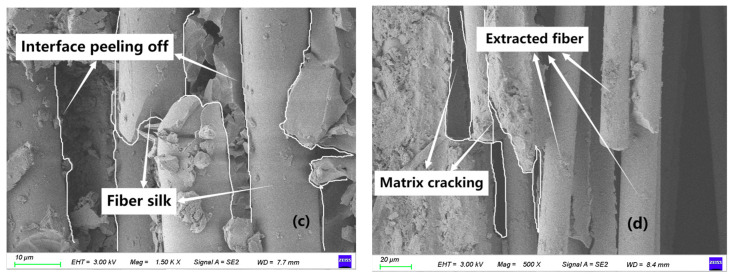
Scanning electron microscope analysis. (**a**) Matrix cracking; (**b**) Interfacial debonding (high magnification); (**c**) Interfacial failure (low magnification); (**d**) Large-area fiber pull-out.

Figure 9a,b reveals the microscopic damage within the bolt-hole shear slip zone. The presence of extensive matrix cracking indicates that the brittle epoxy resin failed under the complex shear-dominated stress state surrounding the bolt hole. This cracking network is the primary pathway for crack propagation, which eventually leads to the macroscopic shear failure observed in Figure 6g.

Additionally, fiber-matrix interfacial debonding (Figure 9c,d) suggests weak adhesion, which facilitates crack deflection along the interface and contributes to the overall loss of structural integrity through mechanisms such as fiber pull-out. The phenomenon of fiber pull-out (rather than fiber fracture) dominates the failure surface, indicating that the shear stress exceeded the fiber-matrix interfacial strength before the fiber tensile strength was reached. This is a common failure mechanism in shear-dominated composites.

### 2.4. Load-Displacement Curves

#### 2.4.1. Load-Displacement Curves for Single Lap Joint Specimens

**Figure 10:**
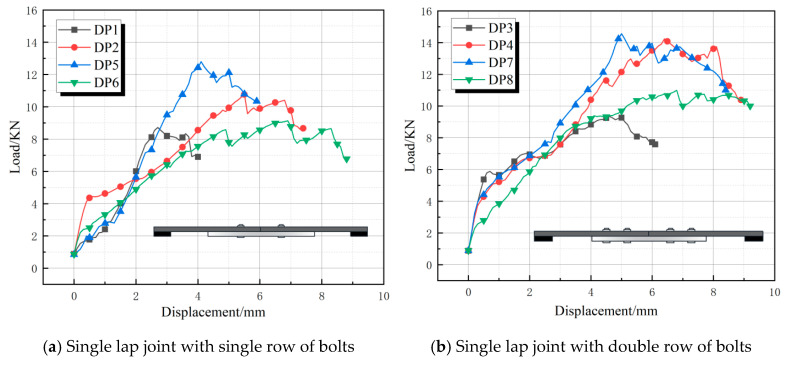
Load-displacement curve for single lap joint.

The load-displacement curves for single lap butt joints (DP1–DP8) are shown in Figure 10. All eight curves exhibited significant fluctuations and a slow rise, followed by a gradual decline in a multi-step oscillation pattern after reaching the ultimate load value [27]. This behavior is attributed to progressive extension of the shear slip zone, causing matrix cracking and fiber failure. The slope of the load-displacement curves for the double-row bolt specimens (DP3, DP4, DP7, DP8) was greater than that of the single-row bolt specimens. This reflected their higher stiffness, tighter joint fit-up, and improved bolt preload effectiveness. Increasing the bolt count enhanced load distribution uniformity. The double-row configuration delayed localized damage accumulation through multiple load-transfer paths, while the single-row assembly experienced concentrated loading that preferentially initiated cracks in vulnerable areas (e.g., end bolt holes). Additionally, enhanced friction in double-row bolt connections further inhibited slippage, thereby improving deformation capacity. The ultimate load values for DP4 and DP7 were 14.22 kN and 14.67 kN, respectively, while DP3 and DP8 (also double-row specimens) reached 9.18 kN and 10.20 kN, respectively. This indicated that factors beyond the number of bolts contributed to the ultimate load.

#### 2.4.2. Load-Displacement Curves for Lap Joint Specimens

**Figure 11:**
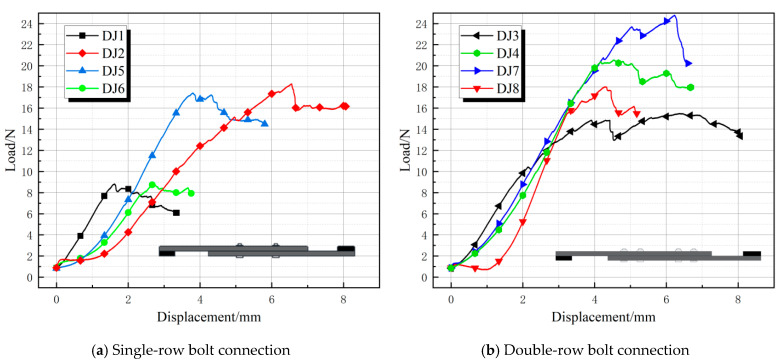
Lap joint load-displacement curve.

Figure 11 shows the eight sets of load-displacement curves for the lap joints (DJ1–DJ8). Compared to single lap joints, the load-displacement curves of the lap joints exhibited a steeper slope during the initial loading phase, with peak loads ranging from 8 to 24.8 kN. The curves rose smoothly to the peak and then exhibited minor fluctuations, and no significant decrease was observed as displacement increased. This indicates that lap specimens exhibit better deformation capacity after reaching the failure load. The double-row bolt groups (e.g., DJ3, DJ4) exhibited a gradual decline after the peak, with peak displacements of 6–8 mm, indicating that they achieve ductile failure through bolt cooperative sliding and plate plastic deformation [28]. In contrast, the four-bolt groups (e.g., DJ2, DJ5) showed a sharp drop after the peak, with peak displacements of only 4–7 mm, exhibiting typical brittle failure (rapid peeling at the lap joint interface). DJ1 and DJ6 had the lowest ultimate loads among the eight specimens, both around 9 kN, which may be due to the use of only four bolts and a preload torque of 6 N·m. Although DJ1 and DJ6 had different bolt end distances and connection lengths, these differences did not significantly affect their ultimate loads. DJ5 and DJ6 also exhibited the same phenomenon, but due to the higher preload torque and larger bolt diameter, the ultimate load of DJ5 and DJ6 reached 17 kN.

#### 2.4.3. Load-Displacement Curves for Double Lap Joint Specimens

**Figure 12:**
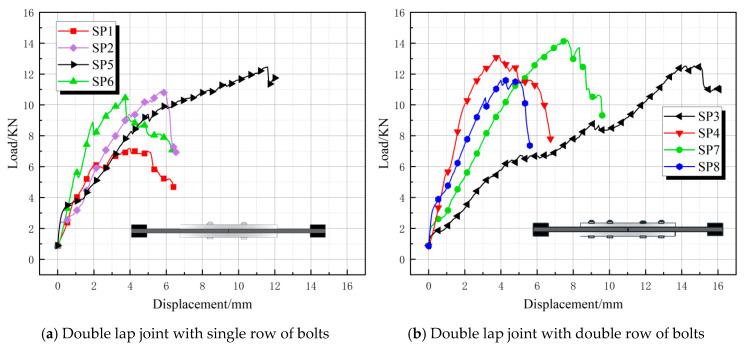
Load-displacement curve for double lap joint.

Figure 12 shows the eight sets of load-displacement curves for the double-lap joint specimens (SP1–SP8). The SP specimen curves generally exhibited an “n” shape, with a distinct and rapid decline segment after reaching the ultimate load value. This indicates that upon reaching the ultimate load, SP specimens sustained load less effectively than DJ specimens. The load values for double-row bolts were significantly higher than those for single-row bolts. SP4 and SP7 were the two groups with the highest load values, exceeding 13 kN. SP7’s peak displacement was approximately 7 mm (second only to SP5), which was far greater than SP4’s. SP5 had a peak displacement of 11 mm—approximately 4 mm larger than SP7—while its load-bearing capacity was only about 2 kN lower than SP7’s. Therefore, considering both load-bearing capacity and deformation capability, the SP5 configuration is optimal. SP6 exhibited the highest initial stiffness, with load rapidly rising to peak (>11 kN) but minimal displacement (<4 mm), followed by an abrupt drop after peak. Its high stiffness indicated tight bolt connection, while the brittle drop suggested sudden failure. This demonstrates that configurations with smaller-diameter bolts and lower preload torque made the BFRP matrix more prone to cracking. Reduced friction contribution to load-bearing capacity also accelerated fiber failure. The curves for low preload torque groups (DJ1, DJ3, DJ6, DJ8) exhibited sharper, finer fluctuations. Low preload torque exacerbated delamination propagation, causing load instability [29].

### 2.5. Parameter Sensitivity Analysis

Sensitivity analysis typically evaluates how structural responses vary with changes in design parameters. The adequacy of structural parameter selection was directly impacted by connection safety and cost-effectiveness [30]. Key parameters across different connection configurations included the number of bolts, bolt end distance, bolt preload, connection length, bolt material, and bolt diameter. Investigating the effects of these parameters on BFRP panel load-bearing performance was critical for optimizing photovoltaic mounting system design. This study employed range analysis and analysis of variance (ANOVA) to comprehensively determine optimal factor levels [31].

**Figure 13:**
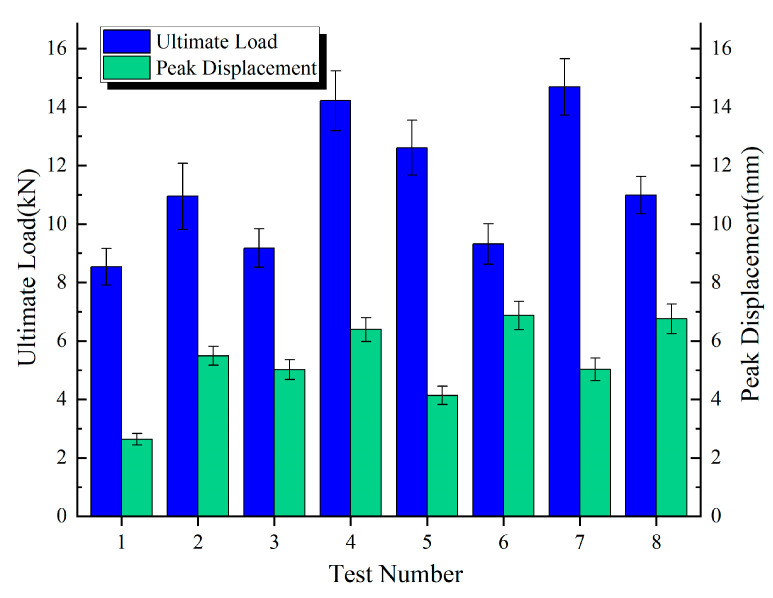
Mean ± Std for DP specimens.

**Figure 14:**
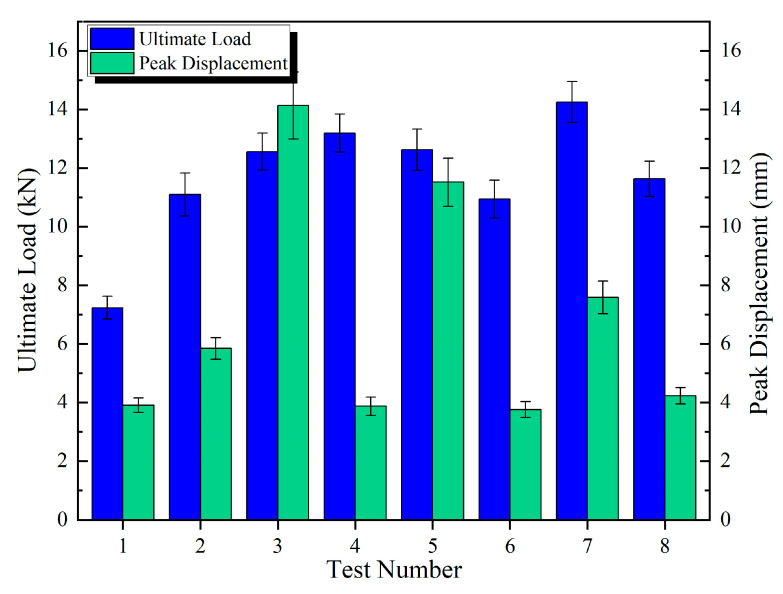
Mean ± Std for SP specimens.

**Figure 15:**
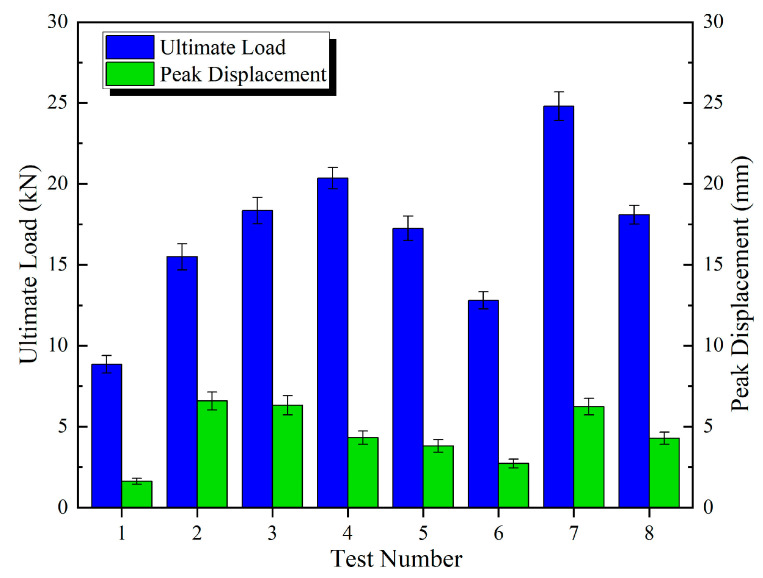
Mean ± Std for DJ specimens.

The discrete metrics for the ultimate load and peak displacement under different connection configurations are summarized in Table 6, Table 7 and Table 8 and Figure 13, Figure 14 and Figure 15.

#### 2.5.1. Analysis of Ultimate Load and Peak Displacement Response Values

**Figure 16:**
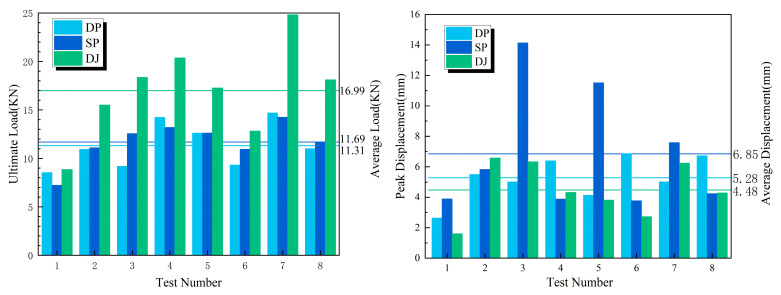
Ultimate load and peak displacement response values.

Figure 16 presents the ultimate load, peak displacement response values, and group averages for the three connection configurations. The average ultimate loads for the DP, SP, and DJ configurations were 11.31 kN, 11.69 kN, and 16.99 kN, respectively. The DJ configuration demonstrated the highest ultimate load, exhibiting increases of 45.3% and 50.2% compared to SP and DP. The average peak displacements for DP, SP, and DJ were 5.28 mm, 6.85 mm, and 4.48 mm, respectively. The SP configuration achieved the highest peak displacement, exceeding DJ and DP by 52.9% and 29.7%, respectively. Based on the overall bar chart profiles, the eight ultimate load values for SP and DP configurations exhibited minimal fluctuations, indicating enhanced design robustness and reduced sensitivity to construction variability.

**Figure 17:**
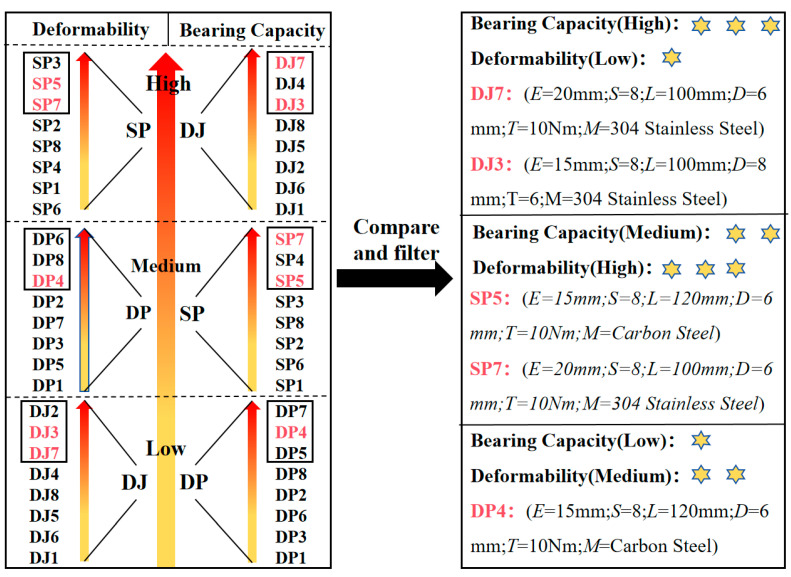
Comparison chart of comprehensive performances of test specimens (The performance levels—low, medium, and high—are intuitively assessed using 1 to 3 stars.

As illustrated in Figure 17, the average ultimate load (bearing capacity) and peak displacement (deformation capacity) for the three configurations are arranged in two columns following a “low, medium, strong” sequence. The left column displays the inter-group bearing capacity ranking DJ > SP > DP, while the right column shows the deformation capacity ranking SP > DP > DJ. Within each column, the top three specimens in bearing capacity and peak displacement are then identified. Finally, specimens appearing in the top three for both bearing capacity and deformation capacity are designated as those with optimal comprehensive performance.

#### 2.5.2. Range Analysis

Range analysis is a method used to evaluate the influence of different factors on system or model outcomes by comparing output variations across factor levels [32]. To quantify the degree to which each factor affects the ultimate load and peak displacement [33], the data were divided into two groups based on the number of levels. The absolute value of the difference between the means of these groups was calculated as the range (R). The results are presented in Table 9 and Table 10.

**Figure 18:**
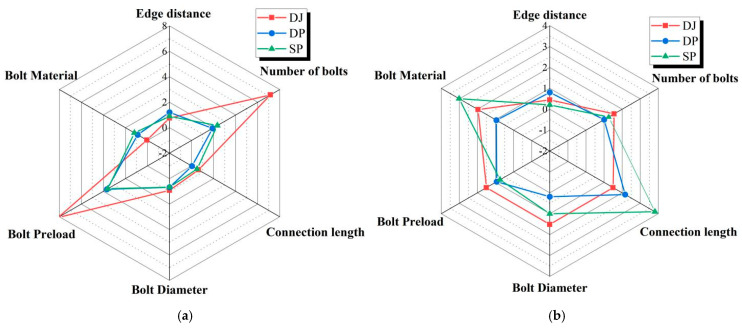
Influence degree of various factors on ultimate load (**a**) and peak displacement (**b**).

As shown in Table 9 and Figure 18a, the number of bolts and bolt preload exerted significant influence on the ultimate loads of all three connection configurations. In contrast, bolt material, connection length, and bolt diameter exerted negligible effects on the ultimate loads. Under these factors, the ultimate load of the lap joint (DJ) reached nearly 8 kN, substantially exceeding those of the single lap joint (DP) and double lap joint (SP). The largely overlapping radar chart profiles for DP and SP indicate that the ultimate loads of these two configurations were similarly affected by each factor.

As shown in Table 10 and Figure 18b, connection length and bolt material had the greatest influence on the peak displacement of double lap joints (SP), with values reaching 3–4 mm. With the exception of bolt end distance, the remaining five factors affected the peak displacement of lap joints (DJ) within a range of 1–2 mm. For single lap joints (DP), peak displacement was most significantly influenced by connection length, reaching 2.166 mm.

#### 2.5.3. Analysis of Variance (ANOVA)

Analysis of variance in orthogonal experiments is a method applying analysis of variance principles to data obtained from orthogonal experimental designs. This approach quantifies whether the effects of various factors on the ultimate load values of different connection configurations are statistically significant (Table 11, Table 12 and Table 13).

## 3. Calculation Model for the Load-Bearing Capacity of Bolted-Connection BFRP Plates

### 3.1. Friction Coefficient Test

As evidenced by the sensitivity analysis in Section 2, the bolt preload torque is a primary influencing factor and directly governs the magnitude of the frictional force. Hence, the accurate determination of the coefficient of friction at the connection interface is imperative. The friction coefficient between BFRP plates and between BFRP plates and aluminum alloy plates was measured using a reciprocating friction and wear testing machine, providing effective support for the accuracy and stability of the load model.

#### 3.1.1. Testing Method

**Figure 19:**
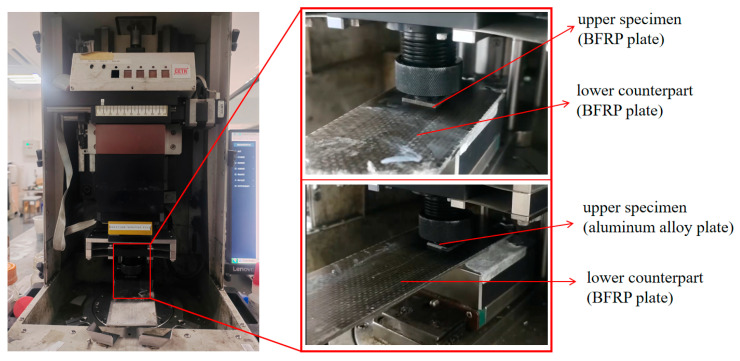
Reciprocating sliding friction test.

The friction tests were conducted in accordance with ASTM G133 [34] standard using a UMT-3 reciprocating tribometer under ambient conditions (23 ± 2 °C, 50 ± 5% relative humidity). A normal load of 150 N was applied with a reciprocating stroke length of 25 mm at a frequency of 1 Hz. The total test duration was 0.5 h (Figure 19). In the first group, an aluminum alloy plate (10 × 10 mm) served as the upper specimen, while a fiberboard sample (50 × 30 mm) was used as the lower counterpart. Prior to testing, both specimens were thoroughly cleaned with acetone and lint-free wipes to remove surface oils and contaminants. The sliding direction was aligned with the longitudinal fiber orientation of the fiberboard, i.e., parallel to the loading direction used in tensile tests.

#### 3.1.2. Friction Test Results

**Figure 20:**
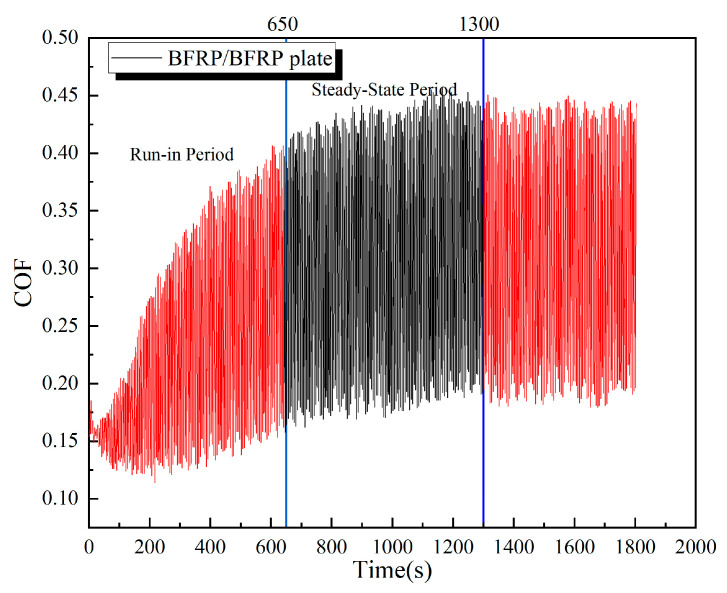
COF time curves for BFRP-to-BFRP.

**Figure 21:**
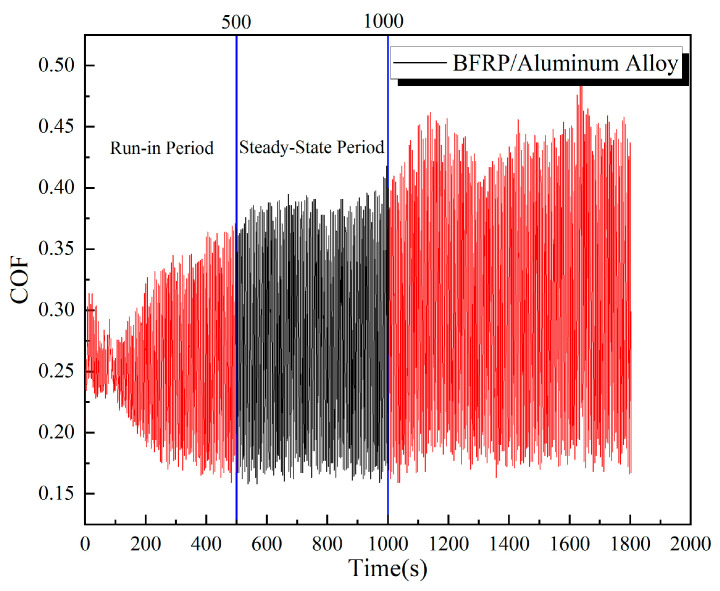
COF time curves for BFRP-to-aluminum alloy.

Figure 20 shows the evolution of the coefficient of friction (COF) with time for the BFRP-BFRP plate pair under a load of 150 N. The curve can be characterized by a brief run-in period (approximately the first 650 s), followed by a relatively steady state. Between approximately 650 and 1300 s of testing, the contact surfaces underwent a run-in period and reached a state of dynamic equilibrium, during which the wear rate stabilized. The friction coefficient curve became relatively smooth, oscillating around a mean value. Figure 21 shows the friction coefficient (COF) versus time curve for the BFRP plate sliding against the aluminum alloy plate. The steady-state period was observed from approximately 500 to 1000 s.

The average coefficient of friction and its standard deviation corresponding to this steady-state period are summarized in Table 14.

### 3.2. Bearing Capacity Calculation

**Figure 22:**
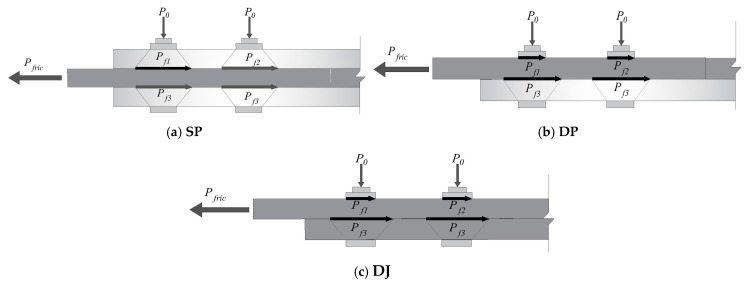
Schematic diagram of friction forces on BFRP panels with three types of connections.

In this study, the bolt preload torque was applied using a calibrated torque wrench. The torque coefficient K was taken as 0.3, a value recommended in references for dry, unlubricated carbon steel/stainless steel bolted connections with comparable surface conditions [35,36]. It is acknowledged that the value of K can vary significantly depending on surface treatment, lubrication state, and thread quality, which constitutes a potential major source of scatter in the predictions of the proposed model. To ensure consistency, all bolts, nuts, and washers were sourced from the same batch and an identical tightening procedure was followed for all specimens (e.g., hand-tightened to snug tightness, followed by torque wrench application at a steady rate) to minimize variability in the achieved preload [37]. Experimental observations showed that the failure modes were exclusively end-distance shear slip (i.e., plate slip failure at bolt hole edges due to shear stress) and bolt hole extrusion failure. No other failure modes such as bolt fracture or static interface fracture were observed, consistent with studies involving similar material combinations. Figure 22 presents simplified friction calculation diagrams for the three connection configurations. The calculation model adopts the following simplifying assumptions.

(1) The shear contribution of the bolt shank is neglected (failure occurs exclusively in the base material);
(2) Preload distribution is assumed to be uniform;
(3) Failure progression begins at the first row of bolts and propagates gradually until complete connection failure occurs.

Total load-bearing capacity of BFRP panel bolt connections:

(1) $$P_{u}={\alpha P}_{\mathrm{fric}}+min(P_{sh},P_{br})$$

In this equation, α is the friction force decay coefficient (0.8 for SP and DJ, 0.75 for DP); $P_{\mathrm{fric}}$ is the inter-panel friction force generated by the bolt preload torque (N); $P_{sh}$ is the bolt hole shear load-bearing capacity (N); and $P_{br}$ is the bolt hole compression load-bearing capacity (N). Inter-panel friction force:

(2) $$P_{f\mathrm{ric}}=n\mu P_{0}$$

where n is the effective number of bolts (contributing to the load-bearing capacity); μ is the interface friction coefficient (0.2547 for SP and DP, 0.3026 for DJ); $P_{0}$ is the bolt preload torque (Nm); d is the bolt diameter (mm); P_0_ is the bolt preload; and P_0_ = 1000 T/Kd, where K is the torque coefficient.

Single bolt shear bearing capacity:

(3) $$P_{sh}=2\tau_{\mathrm{ult}}A_{s}$$

**Figure 23:**
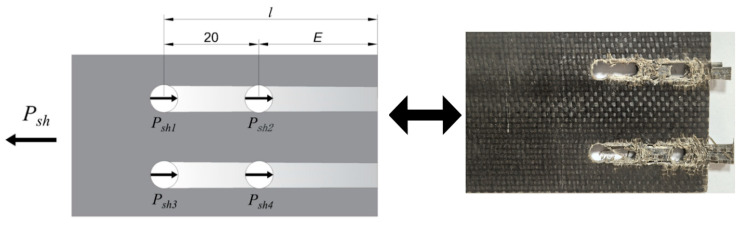
Schematic diagram of shear force on the BFRP plate.

In this equation, $\tau_{\mathrm{ult}}$ is the transverse shear strength of the BFRP plate; as shown in Figure 23, $A_{s}$ is the single bolt shear joint area, and $A_{s}=2lt$, where t is the shear thickness and l is the effective shear length (the distance from the edge of the bolt hole where shear slip begins to the end of the material minus the diameter of the bolt hole along the path).

Since all bolt holes are single-sided compression, the load acts only on one side of the hole, resulting in uneven stress distribution and a higher risk of local crushing. A correction factor K_s_ (with a value of 0.6) is introduced to reflect the strength reduction of single-sided compression relative to double-sided compression [38]; the total bolt hole single-sided compression bearing capacity can be expressed as Equation (4):

(4) $$P_{br}=\;Ksndtf_{br}$$

where $f_{br}$ is the axial compressive strength of the BFRP plate (MPa) and d is the bolt diameter (mm).

Since $P_{br}$/$P_{sh}$ > 1, the total bearing capacity formula is expressed as Equation (5):

(5) $$P_{u}={\alpha P}_{\mathrm{fric}}+min(P_{sh},P_{br})={\alpha P}_{\mathrm{fric}}+P_{br}=\alpha \frac{1000\mu nT}{Kd}+4\tau_{\mathrm{ult}}lt$$

**Figure 24:**
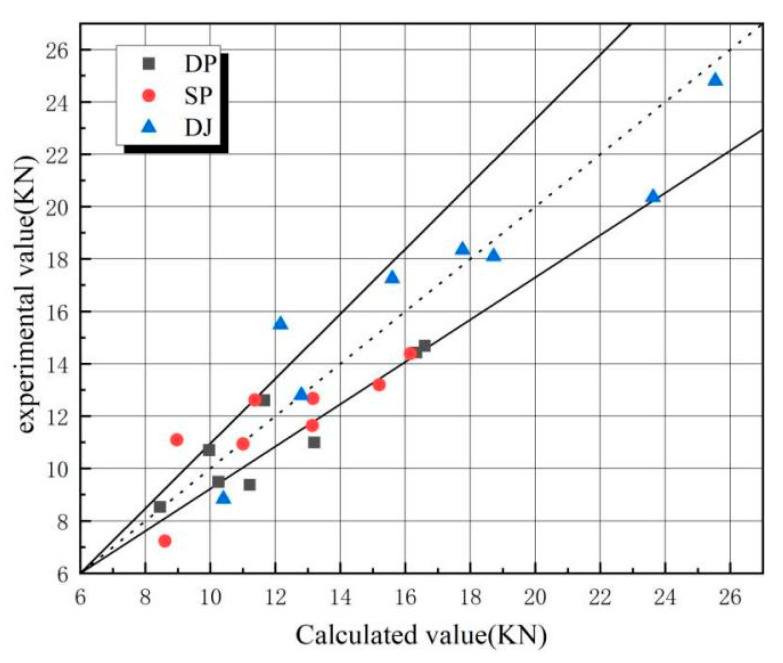
Comparison of calculated and experimental values for ultimate bearing capacity (The dashed line and the two solid lines represent errors of 0% and ±15%, respectively.

Table 15 and Figure 24 compare calculated and experimental ultimate load-bearing capacities for 24 specimens (DP, SP, DJ), with straight lines indicating ±15% error margins. Discrepancies exceeding ±15% in six calculated results versus experimental values may originate from the following.

(1) Variability in BFRP material properties;
(2) Inaccuracies in hole positioning and dimensional tolerances during manufacturing;
(3) Non-uniform load distribution among bolt groups;
(4) Preload relaxation induced by testing vibrations.

**Table 15:** Comparison of calculated and tested ultimate bearing capacity values.

Test Number	DP			SP			DJ		
	Test Value (Mean)	Calculated Value	Error (%)	Test Value (Mean)	Calculated Value	Error (%)	Test Value (Mean)	Calculated Value	Error (%)
1	8.54	8.453	−0.97	7.23	8.612	19.16	8.835	10.431	18.07
2	10.93	9.962	−9.72	11.09	8.973	−19.09	15.492	12.16	−21.51
3	9.37	12.02	21.98	12.56	13.161	4.82	18.345	17.765	−3.16
4	14.23	16.64	13.74	13.19	15.201	15.23	20.355	23.62	16.04
5	12.61	11.16	−11.45	12.62	11.372	−9.86	17.252	15.685	−9.08
6	9.32	10.85	17.78	10.94	11.014	0.71	12.801	12.82	0.15
7	14.69	16.62	11.63	14.25	16.165	13.48	24.802	25.543	2.99
8	10.99	12.09	9.06	11.63	13.141	12.93	18.095	18.720	3.45

### 3.3. Sensitivity Analysis of the Semi-Empirical Model

Table 16 presents the predicted ultimate load values for specimen DJ2 across the practical ranges of μ (0.20–0.30) and α (0.75–0.85). Using the DJ2 specimen as the benchmark, with its experimental value of 15.492 kN, the relative errors were calculated across the μ-α plane. Table 17 displays the relative errors between the benchmark value and the predicted ultimate load values.

### 3.4. Limitations and Applicability of the Model

The proposed semi-empirical model (Equation (5)) is specifically developed for failure modes dominated by shear slip, which was the predominant failure mechanism observed in the present experimental study. This model assumes that the connection fails due to shear along the bolt hole edge and frictional slip between the connected plates, without considering other potential failure modes such as bolt fracture, delamination of the composite laminate, end tearing of the BFRP plate, and combined tension-shear failure.

Therefore, the model is not applicable to connections that exhibit these alternative failure mechanisms. The validity of the model is contingent upon the following conditions.

(1) Shear slip along the bolt hole to the end distance;
(2) Pure tensile loading without significant bending moments;
(3) Accurate and consistent bolt preload application.

Future extensions of this model to other failure modes (e.g., bolt failure, delamination) would require incorporating additional failure criteria and mechanical models such as bolt strength criteria (e.g., tensile and shear strength of bolts), interlaminar shear strength models for delamination, and fracture mechanics-based models for end tearing. Such extensions are beyond the scope of this study but represent a valuable direction for future research.

### 3.5. Comparison with Design Code Requirements

Despite the current absence of national and industry-specific design codes dedicated to BFRP bolted connections, this study validated the engineering applicability of experimental results by referencing evaluation principles from the ASTM D5961 [24] “Standard Test Method for Bearing Response of Polymer Matrix Composite Laminates” and integrating fundamental design requirements for FRP connections specified in “Technical Code for Application of Fiber Reinforced Polymer Composites in Construction Engineering” (GB 50608-2010). Comparative analyses were conducted as follows.

(1) Ultimate Bearing Capacity Comparison

The average ultimate bearing capacities for DJ, SP, and DP connection configurations in this experiment were 16.99 kN, 11.69 kN, and 11.31 kN, respectively. Following the safety factor recommendations (typically 2.5–3.0) for FRP structural connections in GB 50608-2010, if service loads are restricted to ≤1/3 of ultimate capacities as design control criteria, the allowable working loads for DJ, SP, and DP connections would be approximately 5.66 kN, 3.90 kN, and 3.77 kN, respectively. This load-bearing level fully satisfies the force requirements under wind and snow loads in typical photovoltaic support systems (usually single-point load ≤3 kN), demonstrating that BFRP bolted connections possess substantial safety reserves in bearing capacity.

(2) Conformance with Deformation Requirements in Codes

ASTM D5961 emphasizes connection nodes must exhibit adequate deformation capacity to prevent brittle failure. The SP connection demonstrated favorable ductility with an average peak displacement of 6.85 mm. Comparative analysis of test curves reveals both DJ and SP connections maintain certain load-bearing capacity after peak loads with progressive displacement development, aligning with the design principle of “ductile failure prior to brittle failure.” The DP connection, however, exhibits limited displacement capacity (average 5.28 mm) due to bending moment effects, accompanied by local failures such as bolt tilting and embedding. Its application should be strictly restricted in design.

(3) Preload Control Consistency with Standards

This study confirmed bolt preload as a critical factor influencing connection performance. By referencing preload control requirements for high-strength bolts in “Steel Structure Design Standard” (GB 50017-2017), the applied preloading torques (6 N·m, 10 N·m) correspond to reasonable preload ranges after conversion, meeting engineering requirements for anti-loosening and anti-slippage measures.

Through comparative analysis with existing code principles, the following conclusions are drawn: The investigated BFRP bolted connections demonstrate compliance with fundamental engineering requirements in both bearing capacity and deformation performance, and DJ and SP connection configurations exhibit superior performance characteristics and are recommended for primary load-bearing nodes.

## 4. Conclusions

(1) In the lap joint group, stress concentration at the hole edges triggered longitudinal shear crack initiation, along with bolt hole deformation due to compression. Stress concentration at the bolt-hole wall interface caused base material cracking and fiber shear failure. In the single lap joint group, asymmetric loading induced bending moments, leading to bolt tilting and embedment into the hole walls, thereby exacerbating bolt hole compression damage. The double-lap joint’s bilateral constraints suppressed bolt tilting, extended the shear slip path, and resulted in a relatively singular failure mode.
(2) The number of bolts and bolt preload control the ultimate load capacity of DJ and SP connection configurations. In contrast, bolt preload controls the ultimate load capacity of the DP connection configurations. Bolt material and connection length have no significant effect on the ultimate load capacity of these three connection configurations.
(3) Ultimate load ranking: DJ > SP > DP; deformation capacity ranking: SP > DP > DJ. The DJ connection configurations (DJ7 and DJ3 offer the best overall performance) are suitable for critical load-bearing components such as main support structures and wind cable connection points. The DP connection configurations (DP4 offers the best overall performance) are only suitable for non-critical areas with very small loads such as purlin connections and secondary structural connections. Special attention should be paid to the long-term performance degradation risks caused by bolt tilting and embedment issues. The SP connection configurations (SP7 and SP5 offer the best overall performance) are relatively complex. They are suitable for distributed rooftop photovoltaic systems (where people or property exist below) and scenarios where maintenance is inconvenient.
(4) A semi-empirical bearing capacity model (P_u_ = $\alpha \frac{1000\mu nT}{Kd}+4\tau_{\mathrm{ult}}lt$) applicable to slip-dominated failure modes was proposed. The introduction of the friction decay coefficient (α) and unilateral compression correction factor (K_s_) in the formulation demonstrates reasonable accuracy.

## Figures and Tables

**Table 1:** Orthogonal test factors and levels.

Levels	Factors						
	*E* (Bolt End Distance)	*S* (Number of Bolts)	*L* (Connection Length)	*D* (Bolt Diameter)	*T* (Bolt Preload Torque)	*M* (Bolt Material)	Empty Column
1	15 mm	4	100 mm	6 mm	6 N·m	Carbon steel	-
2	20 mm	8	120 mm	8 mm	10 N·m	Stainless steel	-

**Table 2:** Orthogonal array design for structural parameters of bolt joint configurations.

Joint Configurations	Test Number	Factors						
		*E* (Bolt End Distance) (mm)	*S* (Number of Bolts)	*L* (Connection Length) (mm)	*D* (Bolt Diameter) (mm)	*T* (Bolt Preload Torque) (N·m)	*M* (Bolt Material)	Empty Column
DJ	1	15	4	100	6	6	Carbon steel	-
	2	15	4	120	8	10	Stainless steel	
DP	3	15	8	100	8	6	Stainless steel	
	4	15	8	120	6	10	Carbon steel	
	5	20	4	100	8	10	Carbon steel	
	6	20	4	120	6	6	Stainless steel	
SP	7	20	8	100	6	10	Stainless steel	
	8	20	8	120	8	6	Carbon steel	

**Table 3:** BFRP plate material parameters.

Density	Longitudinal Tensile Strength	Longitudinal Tensile Modulus	Elongation at Break	Transverse Shear Strength	Longitudinal Shear Strength	Axial Compressive Strength
20.26 kg/m^2^	649.97 MPa	38.75 GPa	6.12%	60.12 MPa	45.31 MPa	406.00 MPa

**Table 4:** BFRP fiber parameters.

Fiber Content	Ply Count	Manufacturing Process	Thickness	Fiber Volume Fraction	Post-Curing
76.12%	20	Pultrusion	2 ± 0.18 mm	46.20%	-

**Table 5:** Material parameters of 6061 aluminum alloy plates.

Tensile Strength	Yield Strength	Density	Poisson’s Ratio	Elastic Modulus
315.00 MPa	280.00 MPa	2.68 g/cm^3^	0.32	68.80 GPa

**Table 6:** Statistical data summary for DP specimens.

Test Number	Ultimate Load (kN)							Peak Displacement (mm)						
	DP_1_	DP_2_	DP_3_	Mean	Std	Range	CV (%)	DP_1_	DP_2_	DP_3_	Mean	Std	Range	CV (%)
1	7.82	8.92	8.88	8.54	0.58	1.1	6.8	2.41	2.72	2.78	2.64	0.19	0.37	8.2
2	9.65	11.48	11.72	10.93	1.04	2.07	9.5	5.12	5.67	5.68	5.49	0.32	0.56	5.8
3	8.95	9.36	9.73	9.37	0.64	1.28	7.0	4.63	5.21	5.22	5.02	0.34	0.59	6.8
4	13.05	14.68	14.93	14.22	0.94	1.88	6.6	5.92	6.67	6.58	6.39	0.41	0.75	6.4
5	11.58	12.84	13.41	12.61	0.92	1.83	7.3	3.78	4.28	4.35	4.14	0.31	0.57	7.5
6	8.52	9.68	9.76	9.32	0.65	1.24	6.9	6.32	7.25	7.05	6.87	0.47	0.93	6.8
7	13.58	15.12	15.37	14.69	0.90	1.79	6.1	4.58	5.27	5.22	5.02	0.38	0.69	9.6
8	10.25	11.38	11.34	10.99	0.61	1.13	5.5	6.18	7.13	6.95	6.75	0.49	0.95	7.3

**Table 7:** Statistical data summary for SP specimens.

Test Number	Ultimate Load (kN)							Peak Displacement (mm)						
	SP_1_	SP_2_	SP_3_	Mean	Std	Range	CV (%)	SP_1_	SP_2_	SP_3_	Mean	Std	Range	CV (%)
1	6.78	7.45	7.46	7.23	0.39	0.68	5.4	3.62	4.05	4.04	3.9	0.25	0.43	6.4
2	10.25	11.58	11.44	11.09	0.67	1.33	6	5.42	5.98	6.12	5.83	0.48	0.95	8.2
3	11.85	12.78	13.05	12.56	0.60	1.20	4.8	12.85	14.48	15.06	14.13	1.11	2.21	7.9
4	12.45	13.58	13.54	13.19	0.62	1.13	4.7	3.52	3.98	4.11	3.87	0.43	0.85	11.1
5	11.85	12.78	13.23	12.62	0.69	1.38	5.5	10.58	11.85	12.11	11.51	0.77	1.53	6.7
6	10.25	11.05	11.52	10.94	0.64	1.27	5.8	3.45	3.88	3.95	3.77	0.43	0.85	11.4
7	13.45	14.58	14.72	14.25	1.31	2.46	9.2	6.95	7.78	8.01	7.58	0.53	1.06	7
8	10.95	11.85	12.09	11.63	0.57	1.14	4.9	3.91	4.38	4.4	4.23	0.28	0.49	6.6

**Table 8:** Statistical data summary for DJ specimens.

Test Number	Ultimate Load (kN)							Peak Displacement (mm)						
	DJ_1_	DJ_2_	DJ_3_	Mean	Std	Range	CV (%)	DJ_1_	DJ_2_	DJ_3_	Mean	Std	Range	CV (%)
1	8.25	8.95	9.32	8.84	0.54	1.07	6.1	1.40	1.75	1.70	1.62	0.18	0.35	11.1
2	14.58	15.78	16.11	15.49	1.29	2.57	8.3	5.95	6.85	6.95	6.58	0.50	1.00	7.6
3	17.45	18.55	19.05	18.35	0.80	1.60	4.4	5.65	6.55	6.75	6.32	0.55	1.10	8.7
4	19.65	20.45	20.95	20.35	1.67	3.13	8.2	3.85	4.55	4.55	4.32	0.40	0.71	9.3
5	16.45	17.35	17.95	17.25	1.51	3.02	8.8	3.35	4.05	4.00	3.8	0.35	0.69	8.2
6	12.25	12.85	13.30	12.80	0.53	1.05	4.1	2.40	2.90	2.85	2.72	0.27	0.50	9.9
7	23.85	24.95	25.60	24.8	2.23	4.46	9.0	5.65	6.55	6.50	6.23	0.45	0.90	7.4
8	17.45	18.25	18.57	18.09	1.59	3.17	8.8	3.85	4.55	4.45	4.28	0.35	0.70	8.2

**Table 9:** Range analysis results for ultimate load response values.

Bolt Joint Configurations	Range of Ultimate Load R_1_ (kN)					
	Bolt End Distance	Number of Bolts	Connection Length	Bolt Diameter	Bolt Preload	Bolt Material
DJ	0.756	7.153	0.629	0.923	8.108	0.049
DP	1.208	1.901	0.040	0.688	3.681	0.887
SP	0.894	2.342	0.500	0.672	3.594	1.186

**Table 10:** Range analysis results for peak displacement response values.

Bolt Joint Configurations	Range of Peak Displacement R_2_ (mm)					
	Bolt End Distance	Number of Bolts	Connection Length	Bolt Diameter	Bolt Preload	Bolt Material
DJ	0.447	1.557	1.503	1.514	1.503	1.965
DP	0.804	1.006	2.166	0.186	0.935	0.950
SP	0.208	1.256	3.814	1.002	0.750	3.010

**Table 11:** Significance judgment of the DP specimen.

Factors	DP						F (Critical Value)	Significance
	MS	*p*	df (Between Groups)	df (Within Groups)	α	F		
Bolt edge distance	2.588	0.532	1	6	0.05	0.448	5.987	-
Number of bolts	5.600	0.283				4.021		-
Connection length	0.003	0.983				0.002		-
Bolt diameter	18.214	0.047				6.350		*
Bolt preload	18.00	0.143				13.546		*
Bolt material	0.520	0.780				0.842		-

“-“: not significant; “*”: significant;

**Table 12:** Significance judgment of the SP specimen.

Factors	SP						F (Critical Value)	Significance
	MS	*p*	df (Between Groups)	df (Within Groups)	α	F		
Bolt edge distance	0.055	0.940	1	6	0.05	0.882	5.987	-
Number of bolts	42.780	0.009				6.671		*
Connection length	5.198	0.395				0.263		-
Bolt diameter	0.240	0.863				0.464		-
Bolt preload	17.694	0.031				12.889		*
Bolt material	1.530	0.645				1.460		-

“-“: not significant; “*”: significant;

**Table 13:** Significance judgment of the DJ specimen.

Factors	DJ						F (Critical Value)	Significance
	MS	*p*	df (Between Groups)	df (Within Groups)	α	F		
Bolt edge distance	6.250	0.629	1	6	0.05	3.156	5.987	-
Number of bolts	19.440	0.935				51.302		*
Connection length	0.005	0.989				0.876		-
Bolt diameter	0.240	0.928				0.256		-
Bolt preload	93.872	0.034				65.780		**
Bolt material	0.255	0.450				0.002		-

“-“: not significant; “*”: significant; “**”: highly significant.

**Table 14:** Two types of friction coefficient test values.

	Mean Friction Coefficient	Standard Deviation	Minimum Value	Maximum Value
μ_1_ The friction coefficient between BFRP plates	0.3026	0.093	0.162	0.442
μ_2_The friction coefficient between BFRP plate and aluminum alloy plate	0.2547	0.081	0.158	0.418

**Table 16:** Ultimate load value of the DJ2 specimen on the μ-α plane.

α\μ	0.20	0.22	0.24	0.26	0.28	0.30
0.75	13,946.67	14,613.33	15,280.00	15,946.67	16,613.33	17,280.00
0.77	14,124.44	14,808.89	15,493.33	16,177.78	16,862.22	17,546.67
0.79	14,302.22	15,004.44	15,706.67	16,408.89	17,111.11	17,813.33
0.81	14,480.00	15,200.00	15,920.00	16,640.00	17,360.00	18,080.00
0.83	14,657.78	15,395.56	16,133.33	16,871.11	17,608.89	18,346.67
0.85	14,835.56	15,591.11	16,346.67	17,102.22	17,857.78	18,613.33

**Table 17:** Relative errors on the μ-α plane for specimen DJ2 against the benchmark value.

α\μ	0.20	0.22	0.24	0.26	0.28	0.30
0.75	−10.0%	−5.7%	−1.4%	2.9%	7.2%	11.5%
0.77	−8.8%	−4.4%	0.0%	4.4%	8.8%	13.2%
0.79	−7.7%	−3.1%	1.4%	5.9%	9.4%	15.0%
0.81	−6.5%	−1.9%	2.8%	7.4%	11.0%	16.7%
0.83	−5.4%	−0.6%	4.1%	8.9%	12.6%	17.4%
0.85	−4.3%	0.6%	5.5%	10.4%	13.2%	19.1%

## Data Availability

The original contributions presented in this study are included in the article. Further inquiries can be directed to the corresponding author.

## Abbreviations

The following abbreviations are used in this manuscript.

BFRP	Basalt fiber-reinforced polymer
DJ	Lap joint
DP	Single lap joint
SP	Double lap joint
